# Unlocking the potential: m6A-RNA methylation in severe epidermolysis bullosa simplex

**DOI:** 10.1042/BSR20253141

**Published:** 2025-07-22

**Authors:** Dario Leonardo Balacco, Benjamin J. Hewitt, Ajoy Bardhan, Lisa M. Shriane, Manrup Hunjan, Robyn Hickerson, Adrian H. M. Heagerty, Iain L. Chapple

**Affiliations:** 1 School of Health Sciences, College of Medicine and Health, The University of Birmingham, Birmingham, United Kingdom; 2 Institute of Inflammation and Ageing, University of Birmingham, Birmingham, United Kingdom; 3 Adult Epidermolysis Bullosa Unit, Department of Dermatology, University Hospitals Birmingham NHS Foundation Trust, Birmingham, United Kingdom; 4 Department of Dermatology, Walsall Manor Hospital, Walsall, United Kingdom; 5 Biological Chemistry and Drug Discovery Unit, School of Life Sciences, University of Dundee, United Kingdom; 6 Birmingham Dental Hospital, Birmingham Community Health NHS Foundation Trust, Birmingham, United Kingdom; 7 Birmingham NIHR BRC in Inflammation, Birmingham, United Kingdom

**Keywords:** epidermolysis bullosa, epitranscriptomics, m6A, mRNA methylation, RNA biology

## Abstract

Epidermolysis bullosa simplex (EBS) is a rare genetic disorder, resulting from mutations in keratin 5 and keratin 14 (KRT14), and is characterised by skin fragility, herpetiform blistering, and the development of confluent palmoplantar keratoderma and nail dystrophy. Inflammation, pain and itch are the most common complications of severe EBS. However, pathophysiological mechanisms remain poorly characterised at a molecular level. Recently, RNA N6-methyladenosine (m6A) nucleotide modification has been implicated in several cutaneous physiological processes, including epidermal differentiation, inflammation, adaptive immune responses, host–pathogen interactions, wound healing and tissue repair. Nevertheless, the role of m6A in EBS has yet to be defined. In this pilot study, we investigated the gene expression of key regulators of m6A, such as writers Methyltransferase-like 3 and 4 (METTL3 and METTL14), readers YTH domain-containing proteins (YTHDC1, YTHDC2, YTHDC3) and YTH domain-containing family proteins ( YTHDF1 and YTHDF2) and erasers fat mass and obesity-associated (FTO) and alkB homolog 5 (ALKBH5), as well as total RNA m6A levels in the EB keratinocites cell line (KEB-7) derived from a patient with severe EBS, carrying the KRT14 R125P mutation. NEB-1 cells, derived from a healthy donor, were employed as controls. RNAseq and quantitative RT-PCR demonstrated up-regulation of the writer METTL14, while FTO was down-regulated. Moreover, the total RNA m6A colorimetric assay reported higher levels of m6A in severe EBS cells (KEB-7). Additionally, increased expression of the reader of YTHDC1 suggests a dysregulation of downstream pathways. These findings suggest a potential role for m6A in determining complications in severe EBS; however, its role and effects need to be fully elucidated.

## Introduction

Epidermolysis bullosa (EB) represents a broad group of rare genetic skin disorders with an incidence of 19.6 per million births and is characterised by epithelial detachment and blistering upon minimal mechanical trauma [1]. A heterogeneous phenotypic spectrum has been described including persistent blistering, inflammation, delayed re-epithelisation, abnormal wound healing, infection, chronic ulceration, scarring, contractures, pseudo-syndactyly and a predisposition to aggressive squamous cell carcinoma (SCC). Extracutaneous manifestations include possible involvement of the trachea, oesophagus, eyes, nails and oral cavity. Consequently, morbidity is significant, and in some cases, early death occurs [2–4]. Individuals can be mildly or severely affected; however, genotype–phenotype (G–P) correlation is highly complex due to the involvement of one of the 16 different structural and regulatory genes and their inheritance pattern, which may differ for the same gene depending on the mutation. Moreover, mutations in different genes can cause similar phenotypes [2,5–10].

Over 30 EB subtypes have been identified; however, EB is classically classified in four major groups based on genes involved and the plane of cleavage within the epidermis/dermo-epidermal junction: EB simplex (EBS), junctional EB, dystrophic EB and Kindler syndrome (KEB).

EBS is the most common form of EB, accounting for 70% of all cases [4], and is characterised by cleavage through basal keratinocytes provoked by minor trauma, leading to mucocutaneous blistering of varying severity and extent. Mutations in keratin 5 (KRT5) and keratin 14 (KRT14) account for approximately 75% of cases. In addition, plectin, Kelch-like family member 24, dystonin, exophillin 5 and Cluster Differentiation 151 can also be affected.

Severe EBS results from mutations in KRT5 and KRT14 and is characterised by skin fragility, herpetiform blistering, and the development of confluent palmoplantar keratoderma and nail dystrophy [4]. Delayed wound healing may also be seen. Severe EBS is potentially life-threatening in neonates; however, an improvement is usually observed in late childhood. Extracutaneous manifestations include blistering within mucosal tissues, including the oral mucosa and oesophagus. An increased incidence of basal cell carcinoma has also been reported [4]. Inflammation, pain and itch are the most common complications involved in EBS pathogenicity. Nevertheless, the molecular and cellular mechanisms triggered by skin fragility in EB are not yet fully elucidated.

In recent years, emerging evidence suggests that mRNA N6-methyladenosine (m6A) nucleotide modification plays a key role in several cutaneous physiological processes involved in EB pathogenicity, such as epidermal differentiation, inflammation, adaptive immune responses, host–pathogen interactions, wound healing and tissue repair [11–16].

N6-methyladenosine is the most abundant RNA nucleotide modification in eukaryotes and is deposited by the m6A methyltransferase complex (MTC). The multiprotein complex consists of two methyltransferase enzymes, METTL3 and METTL14 termed ‘writers’, and the accessory proteins Wilms’ tumour 1-associating protein, Vir-like m6A methyltransferase associated, zinc finger CCCH-type containing 13 (ZC3H13), RNA-binding motif protein 15 and Cbl proto-oncogene like 1. METTL3 is the catalytically active methyltransferase, whereas METTL14 is inactive but essential for the deposition of m6A, increasing METTL3 efficiency [17].

m6A is deposited onto the whole body of the mRNA and is particularly abundant at the 5′UTR, in long exons, in the last exon in proximity of the stop codon and at the 3′UTR. Sites of m6A methylation are characterised by the motif RRACH (R = G/A, H = A/C/U) [18–20]. Proteins containing the YTH domain can specifically bind m6A and act as ‘readers’ to transduce the epitranscriptome signal. In humans, five readers YTHDF1, YTHDF2, YTHDF3, YTHDC1 and YTHDC2 were identified [21–24]. Additional proteins not containing the YTH domain have been suggested to act as m6A readers, such as human antigen R, insulin-like growth factor 2 mRNA-binding proteins and fragile X messenger ribonucleoprotein 1. Moreover, ‘eraser’ proteins, such as fat mass and obesity-associated (FTO) and alkB homolog 5 (ALKBH5), have been identified as m6A mRNA demethylases, suggesting the reversibility and dynamicity of m6A mRNA methylation [25,26].

The role of m6A in the regulation of RNA metabolism and cellular and physiological processes has been extensively studied [27]. At a molecular level, m6A positively regulates splicing efficiency and alternative splicing [28–30], alternative polyadenylation [31–34], mRNA nuclear export [35,36], translation [37–40] and mRNA decay [41,42]. Dysregulation of m6A methylosome members is associated with the onset of several cutaneous pathologies, including psoriasis, Merkel cell carcinoma, cutaneous melanoma and SCC [43–48]. In this study, we investigated the expression levels of m6A writers (METTL3 and METTL14), readers (YTHDC1, YTHDC2, YTHDF1, YTHDF2 and YTHDF3) and erasers (FTO and ALKBH5), and overall RNA m6A levels in keratinocytes derived from a patient affected by severe EBS carrying the mutation KRT14 R125P.

## Hypothesis

Considering the key roles of m6A in epidermal homeostasis and its involvement in cutaneous disorders, we hypothesise that m6A RNA methylation is dysregulated in severe EBS and may contribute to determining the severe phenotype.

## Methods

### Cell culture

The cell lines utilised in this study were generated and characterised by Morley et al. [49]. Specifically, our study used the NEB-1 (healthy donor) and KEB-7 (KRT14 R125P) cell lines. The cell lines were maintained at 36.5°C, with 5% CO_2_ in Dulbecco’s modified Eagle’s medium (Gibco, 1195–065) with 25% Ham’s F12 medium (Gibco, 11756–054), 10% Foetal bovine serum (Thermo Fisher, F9665), 0.4 µg/ml hydrocortisone (Sigma-Aldrich, H4881), 5 µg/ml transferrin (Sigma-Aldrich, T6397), 2×10^-11^ mol/l lyothytonine (Sigma-Aldrich, T6397), 5 µg/ml insulin (Sigma-Aldrich, 12643), 10 ng/ml Epidermal Growth Factor (Merk, E4127) and Anti-Anti (Gibco 15240).

### RNA isolation and cDNA synthesis

Isolation of total RNA was performed from 1 × 10^6^ cells using RNAeasy Plus Mini kit (Qiagen, Cat. 74134). RNA was quantified and its quality was assessed using spectrophotometry (Jenway Genova spectrophotometer) (Supplementary Table 1). cDNA was synthesised using SuperScript IV Reverse Transcriptase (Thermo Fisher, Cat. 18090010) following manufacturer’s instructions.

### RNA sequencing and differential gene expression analysis

RNA sequencing was performed by Novogene (U.K) Co. Ltd. using NovaSeq X Plus Series (PE 150, 6 GB of raw data per sample). The messenger RNA library was prepared following poly-A enrichment. The library was checked with a Qubit and by real-time PCR for quantification and bioanalyser for size distribution detection. RNA raw reads were deposited on the Sequence Read Archive (SRA) with accession number PRJNA1079043. Filtered sequences were mapped to the hg38 reference genome using HISAT2 (v2.0.5). Following gene expression quantification, differential gene expression analysis was performed with DESeq (v1.20.0), using the negative binomial distribution as the *P*-value calculation model, Benjamini–Hochberg as the false discovery rate calculation model, and |log2(FoldChange)| ≥ 0.5 and padj ≤ 0.05.

### Quantitative reverse transcriptase PCR (qRT-PCR)

Reaction mixtures comprised 100 µl SYBR Green I (Roche, Cat. 04707516001), 78 µl water (Sigma, Cat. W4502) and 1 µl of forward and reverse primers (Table 1). Reactions were performed with a LightCycler 480 II (Roche, U.S.A.), with reaction conditions shown in Table 2. Crossing point values were generated within LightCycler 480 software via the second derivative method and exported to QBase+ (Biogazelle, Belgium) for analysis using the calibrated normalised relative quantity (CNRQ) method. Five housekeeping genes were assessed with the geNorm algorithm within QBase+, and the three housekeeping genes with the lowest variability were used as reference genes for sample normalisation.

**Table 1:** Primers used for RT-PCR

Gene	Forward (5′-3′)	Reverse (5′-3′)
ALKBH5	AGATTAGATGCACCCCGGTT	CCGGTTCTCTTCCTTGTCCA
FTO	CATGGGGAAAATGGCAGTGA	GATCCCTGCCTTCGAGATGA
METTL14	ACAATCCTGGGAAGACTAAGACT	GCATGAATGAAGTCCCCGTC
METTL3	CAAGGAAACATGCTGCCTCA	AGAATTCTTGCACTTGGGCC
YTHDC1	GTAGTGCCTCCAGAGAACCT	CTGCTTGCACGTCTATCCAC
YTHDC2	CCTGTCACCAATAAAGAGCGT	GCCCACTTGTCTTGCTCATT
YTHDF1	TGACTTTGAGCCCTACCTTACT	GGTCTCCGTTACTGAGCTGT
YTHDF2	CCTTACTTGAGTCCACAGGC	CGTAGACCAAGCAGCTTCAC
YTHDF3	TCAGTACAAAACGGTTCGATTCA	CGCTGCTTCCCCAAGAGAAT

**Table 2:** qRT-PCR reaction conditions

Reaction step	Temperature (°C)	Ramp rate (°C/s)	Hold time (mm:ss)	Cycles
1 – Preincubation	95	4.4	05:00	1
2 – Amplification	-	-	-	45
2a	90	4.4	00:10	-
2b	60	2.2	00:10	-
2c	72	4.4	00:10	-
3 – Melting Curve	-	-	-	1
3a	95	4.4	00:05	-
3b	65	2.2	01:00	-
3c – Acquisition (5 acquisitions/ °C)	65–97	0.11	-	Continuous
4 – Cooling	4	2.2	30:00	1

### Colorimetric m6A assay

For quantification of m6A RNA methylation in samples, we used the m6A RNA Methylation Quantification kit (Colorimetric) (Abcam, Ab185912), following manufacturer’s instructions. Auto strip washer Elx50 (Bio-Tek instruments, inc.) was used, and absorbance was read at 450 nm using a microplate reader ELX800 (Bio-Tek instruments, inc.).

### Statistical analysis and data visualisation

Statistical analysis for quantitative (q)PCR and the m6A colorimetric assay to compare the two groups NEB-1 and KEB-7 was undertaken in R (v.4.0.2). The Shapiro–Wilk normality test (using the shapiro.test package) was used to determine normality of data distribution (*P*>0.05). The F-test (using the var.test package) was used to determine equal variances of the two groups (*P*>0.05). The T-test (using the t.test package) was used to determine significant differences between the two groups. Data were visualised using GraphPad (10.0). Volcano plot of the differentially expressed genes analysis was created using ggplot2 package (v 3.3.6) in R.

## Results

### RNA sequencing and differential gene expression analysis

**Figure 1:**
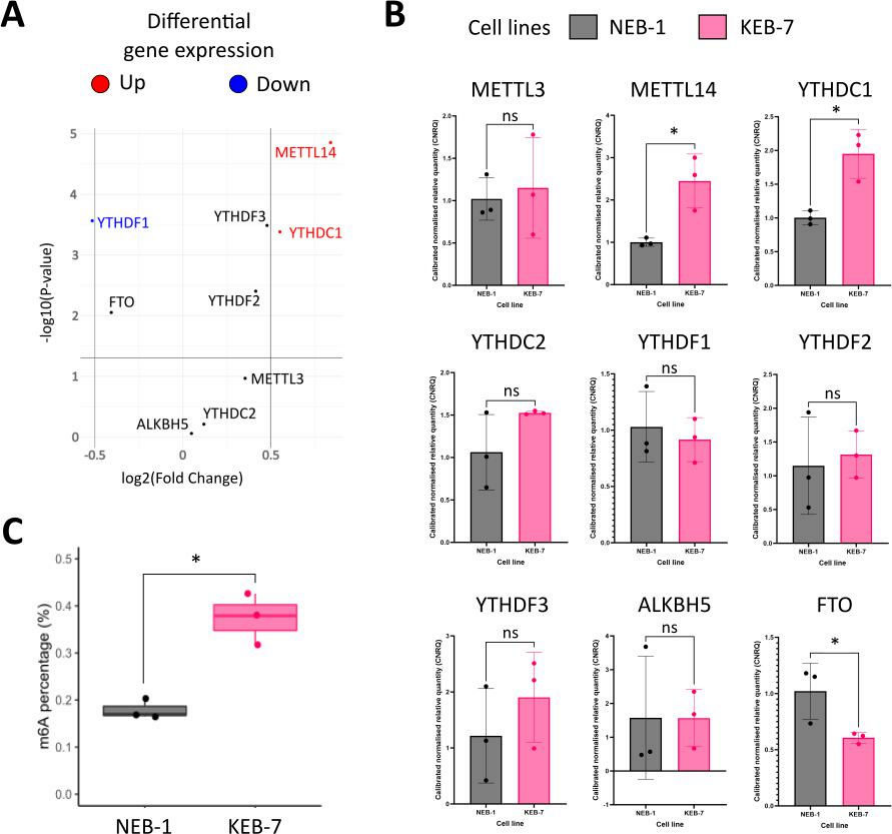
m6A-RNA methylation is increased in severe Epidermolysis bullosa simplex-derived cell line. **(A**) Differential gene expression of m6A writers, erasers and readers between a control cell line NEB-1 (*n*=3) and a severe EBS cell line KEB-7 (*n*=3). The up-regulated genes are highlighted in red, and down-regulated genes in blue. Vertical lines represent the fold change threshold, the horizontal line represents the *P*-value cut-off. (**B**) Reverse transcriptase quantitative PCR of the writers, readers and erasers. NEB-1 and KEB-7 cells are coloured in grey and pink, respectively. (**C**) m6A RNA levels are expressed as m6A percentage. Statistical analysis is indicated with ‘ns’ for non-significant values, and with ‘*’ for *P*-values < 0.05. FTO, fat mass and obesity-associated; ALKBH5, alkB homolog 5.

The mRNA expression levels of the writers, readers and erasers were investigated by mRNA sequencing using Illumina technologies (SRA accession PRJNA1079043). Differential gene expression analysis of mRNA levels revealed that the writer METTL14 was significantly increased with a log_2_(Fold Change) of 0.84, while METTL3 was not increased (Figure 1A, Supplementary Table 2). Moreover, the YTH domain-containing readers exhibited dysregulated expression. Specifically, YTHDF1 was significantly down-regulated with a log_2_(Fold Change) of −0.51, while YTHDF2 and YTHDF3 were up-regulated with a less pronounced increase of a log_2_(Fold Change) of 0.42 and 0.48, respectively (Figure 1A, Supplementary Table 2). YTHDC2 did not show significant changes in expression levels, whereas YTHDC1 was up-regulated with a log_2_(Fold Change) of 0.55. In addition, ALKBH5 was not dysregulated, while the eraser FTO decreased with a log_2_(Fold Change) of −0.4 (Figure 1A, Supplementary Table 2).

### Quantitative RT-PCR

RNAseq findings were validated using qRT-PCR (Figure 1B). In agreement with the RNAseq analysis, the writer METTL3 did not show any significant dysregulation in expression, while METTL14 was significantly increased with an average CNRQ of 2.44. The readers and proteins (YTHDF1, YTHDF2, YTHDF3 and YTHDC2) were not dysregulated; however, expression levels of YTHDC1 were significantly increased with a CNRQ of 1.95. Finally, the eraser ALKBH5 was not dysregulated, while FTO mRNA expression levels were decreased with an average CNRQ of 0.6.

### m6A RNA colorimetric assay

The identification of m6A total RNA levels in NEB-1 and KEB-7 was performed using the m6A RNA colorimetric assay. The analysis revealed that total m6A RNA levels are significantly increased in EB cells (KEB-7) (Figure 1C).

## Discussion

Progress in understanding pathological mechanisms of EB has been slow due to the complexity of G–P correlation. In fact, unexpected manifestations of disease are commonly observed, some milder but some more severe than anticipated. For instance, premature termination codon-causing mutations may result in milder phenotypes than expected [5]. Dysregulation of gene expression at the transcriptional level can also determine disease severity [5].

Due to the low incidence of the disease and the heterogeneity of the phenotype, the study of molecular mechanisms in EB would require the isolation of cells from each patient via skin biopsies. However, isolation and immortalisation of keratinocytes from patients affected by EB is arduous and not always possible; therefore, cell models for EB are scarce. Nevertheless, cell models such as immortalised cell lines are an essential tool in molecular biology, and whilst not perfect, they allow scientists to mimic and study complex biological systems in health and disease. Morley et al. generated stable EB cell lines expressing keratin mutations associated with different severity [49].

In this pilot study, we compared the NEB-1 cell line, derived from a healthy donor, and the KEB-7 cell line, derived from a patient affected by severe EBS carrying the mutation KRT14 R125P [49]. KEB-7 cells form keratin aggregates, a common severe EBS phenotype. However, the impact of KRT14 mutations in inflammation, wound healing and itch in severe EBS remains elusive. Studies in severe EBS keratinocyte cell models linked the formation of KRT14 aggregates to caspase-8 mediated apoptosis [50]. Moreover, chronic inflammation can delay wound healing [51,52]. Interestingly, cultured murine keratinocytes carrying the mutation krt14 R131P, homologous to the human KRT14 R125P, revealed up-regulation of the cytokine thymic stromal lymphopoietin (TSLP), which correlates with pruritus [53]. Additionally, TSLP levels were positively correlated with disease scores for EBS in ~50% of patients [53].

Recent findings delineated a key role of m6A mRNA in several cutaneous processes involved in EB pathogenicity, such as inflammation, immune response, inflammation, wound healing and tissue repair [11–16].

**Figure 2:**
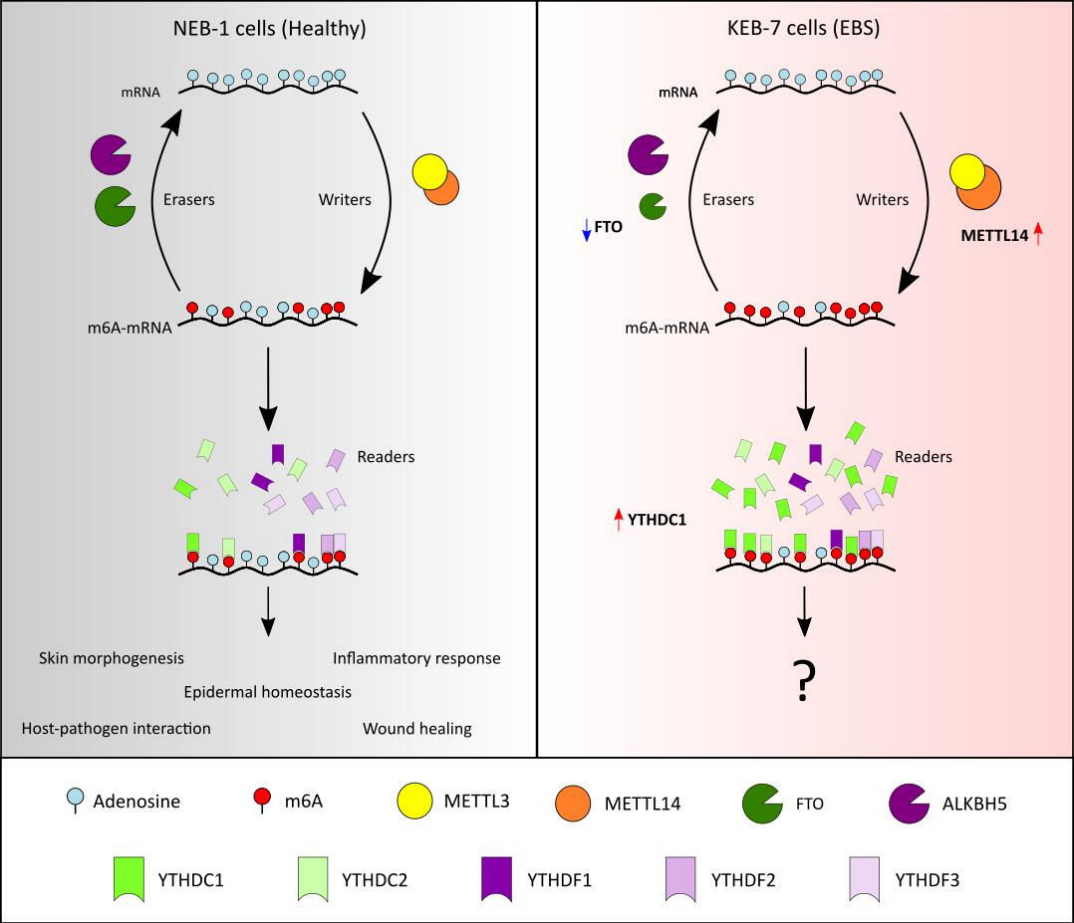
m6A Methylation in severe generalised Epidermolysis bullosa simplex (EBS). In healthy skin cells (NEB-1), m6A methylation is deposited on the mRNA by writers enzyme and is removed by the eraser proteins. Methylation is then decoded by reader proteins, which regulate several physiological processes such as skin morphogenesis, epidermal homeostasis, inflammatory response, host–pathogen interaction and wound healing. In severe EBS cells (KEB-7), we observed an increase in the levels of the METTL14 writer and a decrease in the levels of the eraser FTO, with a consequent increase in overall m6A levels. In addition, the reader YTHDC1 was also found to be increased. The biological function of m6A in EBS remains to be elucidated. m6A, N6-methyladenosine; FTO, fat mass and obesity-associated; ALKBH5, alkB homolog 5.

Our pilot study shows for the first time the dysregulation of m6A methylation in cells derived from a patient affected by severe EBS. Here, using RNA-seq and qRT-PCR, we show that KEB-7, the mRNA expression levels of the writer METTL14 and reader YTHDC1 are up-regulated in severe EBS cells, while expression of the eraser FTO is down-regulated. In turn, the dysregulation resulted in increased m6A RNA levels (Figure 2). While our study identified dysregulation at the transcript level of genes involved in RNA m6A methylation, the relationship between mRNA and protein expression is not linear due to post-transcriptional regulatory processes such as mRNA stability, translation efficiency and localisation. Therefore, further studies are required to determine protein expression levels to confirm dysregulation of proteins involved in m6A methylation.

Huang et al. demonstrated that elevated levels of m6A, as a result of ablation of the eraser ALKBH5 in mice, inhibited keratinocyte migration causing delayed wound healing by boosting mRNA degradation of PELI2, an E3 ubiquitin ligase involved in keratinocyte migration [16]. In addition, higher levels of m6A and higher expression levels of the component of the MTC ZC3H13 were found in keloid tissues and human keloid fibroblasts, linking m6A to hypertrophic scar pathogenesis [54].

Recently, high levels of T helper-17 (TH-17) cytokines were found in lesional skin of patients affected by severe EBS, delineating a key role of TH-17 cells in the pathogenesis of EBS via inflammatory mechanisms [55]. Therapeutic strategies targeting TH-17 cells and IL-17 pathways to improve skin blistering are currently under investigation. An increasing number of studies propose a role for m6A in inflammation and immune response. Yang et al. [56] observed that the circular RNA hsa_circ_000428 plays a key role in skin inflammation in atopic dermatitis, inhibiting M1 macrophage activation in an m6A-dependent manner [56]. Another study suggested that m6A mRNA methylation promotes the activation of dendritic cells, increasing the translation of CD40, CD80 and TLR signalling adaptor Tirap, stimulating T cell activation [57]. In addition, it was shown that IL-6 stimulates METTL14 activity and triggers m6A methylation of TRIM27, which is recognised by the reader IGF2BP2 promoting keratinocytes viability, glycolysis and inflammation [14].

## Future directions

Considering the higher levels of m6A in severe EBS cells found in our study, and dysregulated levels of METTL14, FTO and YTHDC1, we hypothesise a link between m6A and delayed wound healing, inflammation, immune response and itch.

The development of activator or inhibitor molecules of writers, erasers and readers could modulate m6A levels in severe EBS cells, promoting keratinocyte migration and inhibiting the inflammatory response by reducing the regulation of T cells. Further studies are required to investigate the protein expression levels of genes involved in m6A methylation and clarify the role of m6A in EBS. However, our findings, although specific for keratinocytes carrying the mutation KRT14 R125P, highlight the potential for unlocking key aspects of as yet unresolved G–P correlations, with implications for novel therapy development, including modulation of inflammation and infection, improvement of wound healing, skin regeneration, and consequent improvement of wound management and the overall quality of life of patients in both EBS and other EB subtypes.

## Conclusions

In this pilot study, we demonstrate that overall RNA m6A levels in a severe EBS cell line are increased as a consequence of reduced expression levels of the writer METTL14 and increased levels of the demethylase FTO. Increased expression levels of the reader of YTHDC1 suggest a dysregulation of downstream pathways. These findings suggest a potential role of m6A in determining complications in severe EBS; however, the role of m6A in severe EBS still needs to be fully elucidated.

## Supplementary material

Online supplementary table 1

Online supplementary table 2

## Data Availability

Raw RNA-sequencing data supporting the findings presented in this study are available on the Sequence Read Archive under the accession number PRJNA1079043.

## Abbreviations

ALKBH5: alkB homolog 5
CNRQ: calibrated normalised relative quantity
EB: epidermolysis bullosa
EBS: epidermolysis bullosa simplex
FTO: fat mass and obesity-associated
G–P: genotype–phenotype
KRT5: keratin 5
KRT14: keratin 14
MTC: methyltransferase complex
SCC: squamous cell carcinoma
SRA: Sequence Read Archive
TH-17: T helper-17
TSLP: thymic stromal lymphopoietin
ZC3H13: zinc finger CCCH-type containing 13
m6A: N6-methyladenosine
qRT-PCR: quantitative reverse transcriptase PCR
